# The role of next‐generation sequencing in the investigation of ultrasound‐identified fetal structural anomalies

**DOI:** 10.1111/1471-0528.16533

**Published:** 2021-01-03

**Authors:** MD Kilby

**Affiliations:** ^1^ Fetal Medicine Centre Birmingham Women's and Children's Foundation Trust Birmingham UK; ^2^ Institute of Metabolism and Systems Research College of Medical & Dental Sciences University of Birmingham Birmingham UK

**Keywords:** Exome, fetus, genome, sequencing, structural abnormality

## Abstract

Fetal structural anomalies have an impact on fetal mortality and morbidity. Next‐generation sequencing (NGS) may be incorporated into clinical pathways for investigation of paediatric morbidity but can also be used to delineate the prognosis of fetal anomalies. This paper reviews the role of NGS in the investigation of fetal malformations, the literature defining the clinical utility, the technique most commonly used and potential promise and challenges for implementation into clinical practice. Prospective case selection with informative pre‐test counselling by multidisciplinary teams is imperative. Regulated laboratory sequencing, bioinformatic pathways with potential variant identification and conservative matching with the phenotype is important.

**Tweetable abstract:**

Prenatal exome sequencing in fetal structural anomalies yields diagnostic information in up to 20% of cases.

## Tweetable abstract

Prenatal exome sequencing in fetal structural anomalies yields diagnostic information in up to 20% of cases.

## Introduction

Prenatal ultrasound is established as a screening tool in obstetrics and with increasingly high resolution identifies fetal structural abnormalities in approximately 5% of pregnancies.^1^ However, the overall prognosis and long‐term outcome is often variable and dependent upon: (i) the type of principal fetal abnormality, (ii) whether there are additional structural abnormalities and (iii) whether there is an underlying global fetal pathology in the form of a chromosome or gene anomaly. The use of dysmorphology principles may be used to determine the underlying cause from the nature and patterns of fetal anomalies.^2^ Indeed, when fetal abnormalities are identified, further prospective evaluation frequently includes invasive testing to detect whole chromosome aneuploidies (using quantitative fluorescence‐polymerase chain reaction [QF PCR]) and chromosomal microarray analysis (CMA) to identify submicroscopic chromosomal anomalies, collectively referred to as ‘copy number variants’ (CNVs). Overall, QF PCR demonstrates autosomal trisomy in 30%, karyotyping detects pathogenic unbalanced chromosomal rearrangements in 5%, and CMA detects imbalances in up to 6.5% of structural abnormal fetuses.^3^, ^4^ Therefore, approximately 60% of these pregnancies do not have a prospectively identified genomic diagnosis that would guide prognostic information giving and counselling.^5^ A proportion of these ‘unsolved’ cases may be the result of monogenic disease. Information from a family pedigree, previous obstetric history and knowledge of consanguinity may be helpful in the assessment of these pregnancies. Specifically, in fetal medicine, due to the limitations of antenatal imaging, there is an inability to identify subtle dysmorphic features, which hinders the ability to narrow the differential diagnosis. In addition, fetal structural anomalies often vary in expression at ultrasound detection at different gestational ages. These factors limit complete phenotypic information especially in the second trimester, when the initial concern about fetal anomaly typically occurs.

Next‐generation sequencing (NGS), also known as high‐throughput sequencing, is the catch‐all term used to describe a number of different modern sequencing technologies. Whole genome sequencing (WGS) and exome sequencing (ES) provides high resolution testing of DNA down to the single base‐pair level. For WGS, both the non‐coding (introns) and coding (exons) regions on DNA are sequenced without prior selection. For ES, DNA regions of the ‘protein‐encoding’ exons which make up 1–2% of the genome containing >85% of all disease‐causing pathological variants, are captured prior to sequencing.

In WGS, each of the 3 billion bases is sequenced multiple times. Genomic DNA (gDNA) is initially fragmented into a library of small segments that are uniformly and accurately sequenced in parallel reactions. The newly identified strings of bases, called ‘reads’, are then reassembled using bioinformatic software using a known reference genome as a scaffold (a process called alignment). Multiplexing enables large sample numbers to be simultaneously sequenced in a single experiment. In the process, individual ‘barcode’ sequences are introduced to each sample so they can be differentiated during the data analysis.

An alternative to WGS is to sequence only the exomic regions (ES) or part of the exome in a ‘panel’ of (pre)selected genes/variants (see text). To achieve this, a ‘target capture’ step is introduced at the start of the process to select out from the gDNA only those genes or exomic regions to be sequenced.^2^

Information from cohort studies using prenatal WGS is uncommon but is increasing in number. At present relating to the use of prenatal WGS, the literature consists of a single case study identifying disruption of the CHD7 gene in a pregnancy complicated by multiple anomalies (micrognathia, arthrogryposis and polyhydramnios).^6^ In addition, two retrospective cohort series of fetuses with anomalies have been described: one focusing upon increased nuchal translucency in the first trimester^7^ and the other a mixture of prenatal and postnatal cases investigated using ‘low pass’ WGS.^8^ Theoretically, such testing will also detect fetal aneuploidy and copy number variation (which may ultimately bypass the need for prior karyotyping and chromosomal array testing), but bioinformatic filtering and interpretation are more complex, and the methodology is at present more expensive. However, it is likely that in the future, more data will become available on the use of prenatal WGS and this technique has been favoured by the 100,000 Genome Project in the UK. Because of limited data for prenatal WGS, this review will primarily focus upon prenatal diagnosis using ES in the evaluation of fetal structural abnormalities and the expanding evidence base for its utilisation.

This article focuses upon the evidence for prenatal exome sequencing in the prospective evaluation of fetuses with ultrasound identified structural anomalies. It describes the clinical infrastructure required to be in place to optimise pre‐test parental counselling, fetal case selection, laboratory test selection (including bioinformatics pathways and variant calling), the use of exome panels restricted to causative (postnatally defined) known genetic syndromes, the clinical review of ‘potential variants’ and potential ethical dilemmas that may ensue – all this delivered in a rapid turnaround time (TAT) consistent with those adopted for rapid emergency genetic diagnoses in neonatal and paediatric intensive care settings.^9^

## Existing prospective, prenatal exome sequencing studies

In the field of paediatrics, two major prospective cohort studies comprising heterogeneous phenotypes (many associated with childhood neurological sequelae and developmental delay) have demonstrated a diagnostic ‘pathological variant’ yield in up to 30% of cases, aiding understanding and management and allowing discussion of recurrence.^10^, ^11^ In contrast, in obstetrics the majority of studies reported since 2010 describing the use of prenatal exome sequencing in the evaluation of fetal anomalies are small, retrospective single or cohort case series. An informative systematic review including data from peer‐reviewed published papers, published retrospective cohort series and published conference abstracts (containing at least five cases) has been published by a multi‐institutional group of experts.^12^ That publication collated data from 16 ‘citations’ which contained a range of inclusion criteria: fetuses with isolated structural abnormalities, fetuses with multiple fetal abnormalities and those with an increased first trimester nuchal translucency (NT). In addition, the studies included different proportions of fetal anomalies associated with miscarriage, perinatal mortality and termination of pregnancy. Many reported fetus‐only ES testing, whereas others included ‘trio testing’ (fetus, mother and father), a method proven to increase the diagnostic rate.^13^ These 16 studies reported a diagnostic rate that ranged from 6.2^14^ to 80%,^15^ across the studies^6^, ^28^ and documented how inclusion criteria altered the range of positive diagnoses.

**Figure 1:**
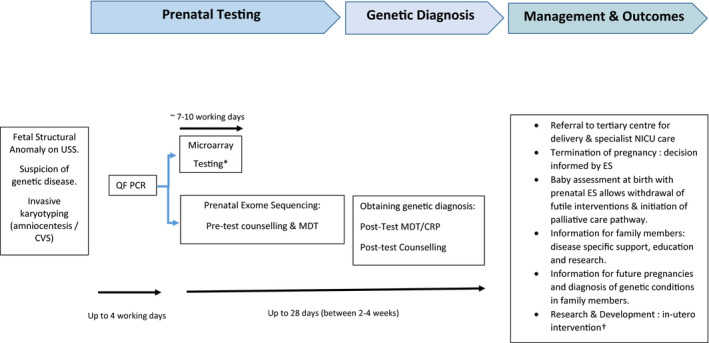
A schematic diagram of the integration of prenatal ES into the current prenatal antenatal diagnostic pathway. Prenatal sonogram (USS), quantitative fluorescence‐polymerase chain reaction (QF PCR) (to exclude autosomic trisomies & monosomy X). MDT, multidisciplinary team meeting; CRP, clinical review panel. *Microarray testing would be initiated in tandem with prenatal ES. ^†^Such as prenatal mesenchymal stem cell transplants by in utero transfusion in OI (BOOST4 study).^65^

However, in early 2019, two relatively large, prospective‐recruited cohort studies (from the UK and USA), targeting ES in fetuses with unselected ultrasound‐detected structural anomalies, were published in *Lancet*.^29^, ^30^ A total of 844 fetal probands, eligible consented ‘fetal trios’, where QF PCR and CMA had previously been performed and was normal, were studied (Figure 1). In 76 probands (9%), a causative pathological genetic variant was identified.

The largest of these was the Prenatal Assessment of Genomes and Exomes (PAGE) study, which performed ES on 610 trios in cases of an identified fetal anomaly and where CMA was negative. Overall, ES provided an additional 8.5% diagnostic yield of pathogenic variants compared with conventional genetic testing. In addition to the pathogenic variants considered to be a direct cause of the relevant fetal structural anomaly, variants of uncertain significance (VUS) and of potential clinical relevance were diagnosed in a further 4% of cases.^29^ The second prospective prenatal series conducted by Columbia University reported an additional diagnostic yield of 10.3% (in 234 trios).^30^ Both these studies gave diagnostic yields that were lower than those reported from retrospective, highly selected case cohort studies.^12^

## Fetal phenotype

The two prospective studies recruited pregnancies in which the fetus had an abnormality identified by ultrasound and the parents opted for antenatal karyotyping (by QF PCR/CMA). Both studies therefore were pragmatic in the identification of an unselected cohort of fetuses with structural malformations in a routine fetal medicine clinical setting.^29^, ^30^ The consequence of this was a heterogeneous mix of anomalies. Both studies recruited a high proportion of fetuses with multiple (>1) structural anomalies. The Petrovski study had a proportion of pregnancies that, although unselected, included cases of cardiac rhabdomyomas and infantile polycystic renal disease (likely to be associated with genetic disease).^30^ In these latter cases, perhaps ‘targeted testing’ could have been utilised.

**Figure 2:**
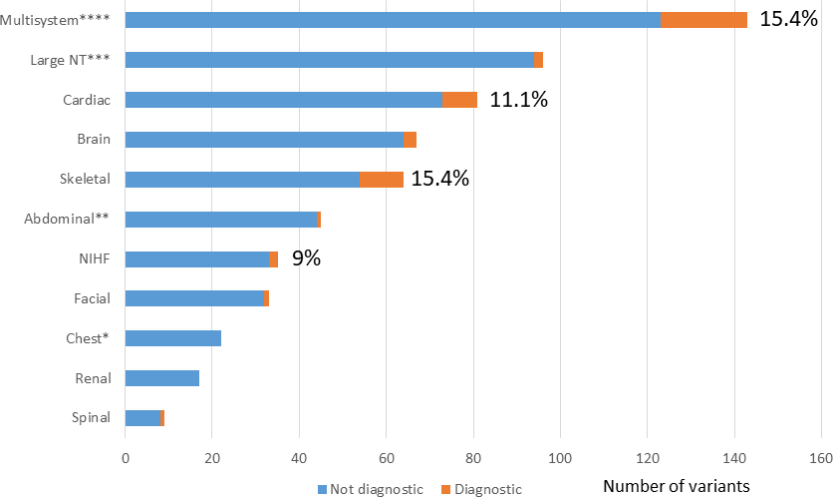
The proportion of diagnostic genetic variants (as a percentage of the total) identified in fetuses with each phenotypic abnormality. *Includes congenital diaphragmatic hernia. **Includes anterior abdominal wall anomalies. ***NT, nuchal translucency (>4 mm). **** Multisystem: >two structural anomalies) (After the PAGE Study).^29^

Both studies found a greater diagnostic rate of pathogenic variants in the presence of multiple congenital anomalies (between 15.4 and 18.9%, respectively).^29^, ^30^ This opens a debate as to whether specifically to select fetuses with specific single anomalies or more than two major abnormalities for testing, and whether testing should be broad or based upon a ‘targeted’ virtual panel dependent on the presenting phenotype. High resolution ultrasound has led to the diagnosis of structural anomalies in the fetus with variable rates of detection,^31^ even with the use of additional modalities such as magnetic resonance imaging.^32^ Subtle dysmorphic features may not be diagnosed and variability of phenotypic expression, incomplete penetrance and varying gestation of presentation make the identification of the fetus at risk of monogenic disorders challenging. The PAGE study identified specific phenotypes that were associated with the highest yield of pathological variants. These included multiple anomalies (15.4%), anomalies of the skeletal (15.4%), cardiac (11.1%) and spinal systems (10%) as well as the presence of non‐immune fetal hydrops (9%) (Figure 2). After correction for multiple testing, the detection of pathological (or likely pathogenic) variants in a fetus with multisystem anomalies was significantly more likely than in a fetus with any single abnormality (*P* = 0.018).^29^ The Petrovski study demonstrated a similar association with structural phenotype; however, renal anomalies (in several fetuses with infantile polycystic disease) were associated with significant monogenic disease (16%).^30^ From the PAGE study, large nuchal translucencies (>4 mm) had a relatively low association with a pathological variant (on subsequent scanning at 20 weeks’ gestation, additional anomalies were identified).^33^ More recently published data evaluating an approach to integrating ES for fetal structural anomalies into clinical practice, also noted the highest yields of pathological variants associated with cardiac and central nervous system, renal, skeletal anomalies, as well as hydrops fetalis and arthrogryposis.^34^, ^35^, ^36^ The NHS England (Genomics) are developing testing criteria based on ultrasound to guide discussions around suitability for prospective prenatal ES (Figure 2). It should also be recognised that mutations in the same gene can cause different prenatal and postnatal phenotypes and may differ with the same underlying genetic pathological variant. A good example of this is the presence of mutations in the histone methyltransferase KMT2D and the demethylase KDM6A genes^37^ associated with Kabuki syndrome. In childhood, this is characterised by classic facial gestalt, multiple organ malformations, abnormal postnatal growth and intellectual disability.^38^ In prenatal life, Kabuki syndrome appears to be associated with nuchal oedema, hydrops fetalis, cardiac and renal malformations, intrauterine growth restriction and associated polyhydramnios.^29^, ^30^, ^39^ Case selection of prenatally identified abnormal fetuses, after genetics review and additional phenotypic information from autopsy, increase diagnostic yield from an index pregnancy.^35^, ^36^ Published postmortem series suggest a yield from ES of up to 30%, reflecting the importance of case selection by detailed, accurate phenotyping (with the inclusion of subtle dysmorphic features).^10^, ^23^, ^36^, ^39^ Obviously, paediatric follow up, and in particular the identification of neurodevelopmental and intellectual disability (not definable prenatally), may also indicate the potential underlying aetiology.^10^, ^11^

## Bioinformatics and interpretation of variants identified using ES

In the PAGE study, sequenced data were assessed for candidate pathogenic variants from a selected group of exons constituting the DDG2P list of 1628 genes, previously used in the DDD study.^11^, ^40^ These were selected as they were identified as rare, protein‐altering variants in which the inheritance pattern of the variant matched that of the gene being assessed for clinical review (by bioinformatic filtering).^29^, ^40^, ^41^ Trio (fetal, paternal and maternal DNA) analysis was used, as it enables filtering of variants according to inheritance patterns and therefore speeds up analysis and reporting. Determining the inheritance pattern can also be helpful in variant classification as an internal validation of the result and can also give information on recurrence risk. These ‘candidate pathological variants’ were then fed into and reviewed by the clinical review panel (CRP), a multidisciplinary group of clinical geneticists, fetal medicine subspecialists, two clinical scientists and a genetic bioinformaticians) who reviewed anonymised variant annotation data and clinical findings using the SAPIENTIA software (version 1.75; Congenica, Cambridge, UK). The CRP reached a consensus view as to the variant classification (i.e. pathological, likely pathogenic, variant of unknown or uncertain significance), likely benign and or not benign, and the likelihood that it was the cause of the fetal phenotype. Using this methodology, an average of 0.4 variants were reviewed per proband (fetus with structural anomaly).^29^

The Petrovski paper used similar but not identical methodology for identification of pathological variants.^30^ Again, they used trio analysis and a previously published framework to allow rapid and efficient identification of de novo and inherited variants.^41^ This focused upon two ‘tiers’ of qualifying genotypes. Tier 1 was associated with the assumption that a relevant genotype would be highly penetrant and be absent from the parents (and controls). Tier 2 was a literature‐motivated screen, which permitted genotypes to be observed at low frequencies among controls (internal and external) which had to have been previously classified as pathogenic on *Clinvar* or Human Gene Mutation databases.^42^, ^43^ Again, potential causative variants were classified by a multidisciplinary conference of specialists to agree genotype/phenotype causation. This methods analysed all genes and also incorporated ‘bioinformatic signatures’, assessing variants in genes that were not yet linked to disease. This resulted in a ten‐fold increase in the variant interpretation burden compared with PAGE (4.8 variants per case versus 0.42 per case requiring manual interpretation by PAGE) (due to the study the whole exome [~20–25 000 genes] was analysed rather than a selected panel of genes [1628 genes in PAGE]), with a limited difference in the overall final pathogenic variant yield.^30^ This demonstrates the need to balance a higher diagnostic yield with higher interpretational burden in a prenatal ES strategy, as well as considering the bioinformatics pipeline adopted. It is also important to realise that causative variant association with phenotype may alter with time and the ‘variant’ list will need to be updated with time. This means that if the fetal trio ES were to be periodically reanalysed (during childhood) every 1–2 years, additional pathological variants might be identified. This has already been recognised in the use of ES/WGS in paediatric datasets.^40^

## Secondary and incidental findings

In clinical practice, variants of uncertain significance are those where pathogenicity is unclear. If fed back to patients, they may cause significant anxiety and make patient decision‐making more complex (especially in the context of a fetal structural abnormality identified in a pregnancy where termination of pregnancy is an option). Parents report that such information is ‘toxic’ and emotional effects may last for a considerable period.^44^, ^45^, ^46^ Therefore, as prenatal ES is used to evaluate congenital malformations in clinical practice, there is a need to register all genetic variants, including VUS and the fetal phenotype in an international registry with comprehensive clinical access. Secondary findings are genetic variants, unrelated to the primary presentation (of the probands) but may be reported if deemed ‘medically actionable’.^12^ In a paediatric setting, the American College of Medical Genetics and Genomic (ACMG) has set out guidelines stating that when offering ES, secondary findings should be reported in 59 genes (in which it is believed that there is clinical evidence that pathological variants may result in disease that may be prevented or treated).^47^ However, with this guidance, there is an exclusion of prenatal ES. The extension of this process to prenatal diagnosis (and the parental samples of trios) has been debated and is controversial.^48^ An example of this would be the potential ES testing of a fetus with a phenotype suggestive of Fanconi anaemia.^49^ Somatic inactivation of the Fanconi anaemia/breast cancer gene (BRCA) pathway accounts for the chromosomal instability of the predisposition of some cancers (breast, bowel and ovary) in the general population.^50^ However, it is recommended that if instituted, it should be at present on a case‐by‐case basis, and careful and detailed pre‐ and post‐test counselling of parents is imperative. In addition, trio ES could also reveal unforeseen issues such as non‐paternity or parental consanguinity, again leading to difficult counselling scenarios.^49^, ^51^, ^52^

## Pre‐test and post‐test counselling

A more than superficial understanding of prenatal ES by parents and its implications for the pregnancy, themselves, their family and future pregnancies is extremely important and cannot be overstated. In the UK, prenatal test counselling in pregnancy has been traditionally the role of screening midwives or specialist genetics counsellors, and when prenatal invasive tests are contemplated it is the obstetrician who usually discusses the procedure and laboratory test with the parents. In North America, this role is often taken by the genetics team, commonly certified specialist genetics counsellors. The pre‐test counselling for potential prenatal ES, therefore, needs to be detailed and intelligible. It must be emphasised that such testing is new and the clinical utility and the detection of specific genetic diagnoses (depending upon the fetal phenotype) are emerging from research studies and will undergo further review as these tests are utilised in clinical practice. There is broad agreement that this should be led by genetic healthcare professionals. However, the challenges of delivering such information to parents (at an emotive time in their pregnancy when a fetal abnormality has been detected) with varying educational, religious and cultural backgrounds and experiences should not be understated.^12^, ^51^, ^52^ It may also raise ethical considerations.^51^, ^52^, ^53^ Our own experience, piloting prenatal ES, is to have a multidisciplinary meeting (to discuss the case study and imaging) and to be conservative about fetal phenotype selection prior to offering the testing.^54^ If the multidisciplinary team (comprising the same group of specialists as used in the PAGE study)^29^ decide that the pregnant woman may be offered such testing, the couple are seen in a multidisciplinary combined fetal medicine/genetic clinic where a repeat ultrasound examination is performed (and previous information from the family pedigree, diagnostic imaging and tests reviewed). The couple then have pre‐test counselling by a fetal medicine subspecialist and a clinical geneticist (Figure 1).^12^, ^54^ I have discussed this clinical pathway and the published data supporting its use in the accompanying presentation given in September 2020 (Video S1).

Once the laboratory‐based ES and potential variants have undergone bio‐informative filtering, a multidisciplinary clinical review panel is essential to discuss the clinical significance of any variant identification and to elucidate the potential likelihood of causation in the associated fetal abnormality or abnormalities. Once a causative genetic variant has been identified, this again needs to be discussed with the parents in a multidisciplinary clinic with the clinical genetic team explaining clinical relevance, potential inheritance and any further testing (especially of family members). In addition, it is not currently possible for either WGS or ES to accurately read every single base pair of every gene and therefore identify every genetic variant. Also, as experience groups and variant matching to phenotype (and pregnancy outcome experience) grows, it is likely that non‐informative variants will be reclassified as pathological in time.^55^ Such complex clinical pathways and infrastructure are starting to be developed in the UK, and multidisciplinary research (through the Optimising EXome PREnatal Sequencing Services [EXPRESS study]) is underway to aid the development of such pathways and to ensure equity of access and high clinical standards across the UK.^56^ However, internationally, different healthcare systems have different methods of delivery of this service.

## Clinical pathway ‘turnaround time’

In the PAGE study, an a priori protocol decision was made only to feedback information on pathological variants at the end of the pregnancy.^29^ The Petroski study (which considered that the results of ES were not intended for use in clinical care) described that it took up to 8 weeks to obtain and interpret results.^30^ Data from paediatric cohorts of critically ill neonates and infants have indicated that rapid ES testing and evaluation is possible (sometimes within 72 hours) and provides clinical benefit and improved decision‐making, aiding developmental and family emotional outcomes.^9^, ^53^, ^57^, ^58^, ^59^ In prenatal diagnosis, there is also a requirement for a relatively rapid TAT to aid informed parental (in terms of consideration of termination of pregnancy) and clinical (in terms of evaluation of active versus palliative newborn care) decision‐making. TAT is affected and influenced by the whole pathway (including the potential need for cell culture to obtain fetal DNA, through the genetic variant bioinformatic filtering and clinical review panel assessment). Further time savings may be made by initiating ES once the results of QF PCR are known and in parallel with CMA analysis (Figure 1). Prenatal TAT therefore can be prioritised when there is a need to obtain a result by a specific gestation time and some centres have achieved this within an average of 14 days (range 7–38 days).^34^, ^35^, ^36^ A recent small retrospective study from the Netherlands indicated that prenatal ES aided parental decision‐making (including decisions on late termination of pregnancy) and aided prenatal/neonatal clinical pathways. Again, on average, TAT was possible within 21 days.^60^. Certainly, parents (pre‐testing) must be aware that test TAT can be of variable duration, with communication between the clinicians and scientists within the laboratory being crucial.

## Cost effectiveness

This will be affected by selection of phenotype, technical considerations such as DNA sequencing, variant interpretation, as well as the infrastructure for pre‐ and post‐test counselling.^12^ The use of ES in suspected monogenic disorders (in children) has been indicated to be increasingly cost‐effective as the benefits of ES data reanalysis, cascade testing in first‐degree relatives, and parental reproductive outcomes are incorporated into modelling.^61^ Our own, rather conservative cost modelling used a decision tree model populated using data from a prospective cohort of women undergoing invasive diagnostic testing. A comparison of four potential testing strategies (after screening for autosomal trisomies) were evaluated using CMA, ES, CMA followed by ES (‘stepwise’), and CMA and ES combined. When ES was priced at GBP 2,100 (EUR 2,407/USD 2,694), performing ES alone prenatally would cost a further GBP 31,410 (EUR 36,001/USD 40,289) per additional genetic diagnosis, whereas stepwise ES would cost a further GBP 24,657 (EUR 28,261/USD 31,627) per additional genetic diagnosis. When ES is priced at GBP 966 (EUR 1,107/USD 1,239), performing ES alone prenatally would cost a further GBP 11,532 (EUR 13,217/USD 14,792) per additional genetic diagnosis, whereas stepwise ES would cost a further GBP 11,639 (EUR 13,340/USD 14,929) per additional genetic diagnosis. The sub‐group analysis suggests that performing stepwise ES on cases indicative of multiple anomalies at ultrasound scan versus cases indicative of a single anomaly, is more cost‐effective compared with using ES alone.^62^ It is likely that these are conservative costs and different healthcare economies will need to evaluate their costs and make decisions as to whether it is possible to implement prenatal exome sequencing within their healthcare systems. Furthermore, this health economic analysis did not take into consideration, detailed and varying neonatal and long‐term paediatric care costs, which are an important consideration.

## The potential implementation in the prenatal diagnostic pathway in England

**Figure 3:**
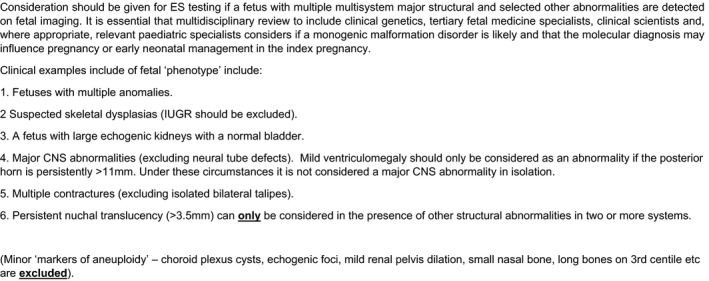
Fetal phenotypes for consideration of prenatal exome sequencing (Rapid Exome Sequencing Service for fetal anomalies testing, NHS England and NHS Improvement, 2020).

In England, a national NHS Genomic Medicine Service infrastructure based around seven laboratory hubs across England is in development. It is envisaged that prenatal case selection will be by a multidisciplinary (tertiary) team, led by a clinic (1) multiple anomalies and structural anomalies where there is a strong suspicion of genetic aetiology (Figure 3). The laboratories charged with providing prenatal ES would use a modified panel of pathological variants identified in the PAGE study (DD‐G2P/PAGE), using a traffic‐light rating and comprising almost 1000 genes that have been chosen after expert review and agreed by NHS Genomic Medicine Service sign off (https://panelapp.genomicsengland.co.uk/panels/478/).^63^ As indicated in the above discussion in this review, the clinical infrastructure for delivery of this prenatal service needs to be conservative but robust. It is, however, also possible that ‘pattern recognition’ of fetal anomalies may lead the MDT to consider panel testing. Such panel tests are specifically designed to interrogate a set of disease‐associated genes. The desirable clinical and laboratory infrastructure required will be informed by prospective, NIHR‐funded research through the EXPRESS study.^56^ However, it is probable that phenotypic inclusion, the pathological variant list and the clinical infrastructure for delivery will remain under continuous review and there is a recognised need for the formal curation of pathological, probable pathological and VUS variants matched to detailed phenotyping within a database such as ClinVar.^42^, ^64^ It is also likely that a strategy for re‐testing will be required, as this has been noted to be important in paediatric testing strategies.^55^

## Conclusion

The implementation of prenatal ES into clinical practice to evaluate the fetus with structural anomalies provides an exciting opportunity to improve delineation of prognosis, provide clinical utility and to understand further the pathogenesis of prenatal genetic disorders. It is also possible that, going forward, the identification of prenatal pathological variants associated with structural anomalies (i.e. severe skeletal dysplasia associated with osteogenesis imperfecta) may provide opportunities for antenatal therapy (such as mesenchymal stem cell transplantation); as in the pan‐EU Brittle Bone Before Birth (BOOSTB4) study.^65^ However, as well as a robust, relatively conservative infrastructure for clinical delivery of this service, there is a need to continue to debate the societal, moral and ethical issues surrounding the implementation of such science so as to provide additional prognostic information to parents while attempting to limit unnecessary emotional burden on parents and society.^51^, ^52^, ^53^, ^54^, ^55^, ^56^, ^57^, ^58^, ^59^, ^60^, ^61^, ^62^, ^63^, ^64^, ^65^, ^66^

### Disclosure of interests

MDK was an investigator and grant holder as part of the PAGE study. This represents research commissioned by the Health Innovation Challenge Fund (HICF‐R7‐396), a parallel funding partnership between the Department of Health and the Wellcome Trust. The views expressed in this publication are those of the author and not necessarily those of the Department of Health or the Wellcome Trust. MDK is also a member of the RCOG Genomics Taskforce, the RCOG representative of the Joint Committee on Genomics in Medicine (joint committee of the Royal College of Physicians, Royal College of Pathologists, Royal College of Paediatricians & Child Health, Royal of Obstetricians and Gynaecologists) and a member of the Fetal Group of the British Society of Genetic Medicine. A completed disclosure of interest form is available to view online as supporting information.

### Contribution to authorship

MDK had the idea for this review and planned, researched, wrote and revised this article.

## Supporting information


**Video S1.** PowerPoint and video presentation.

Supplementary Material

## References

[1] Dolk H , Loane M , Garne E . The prevalence of congenital anomalies in Europe. Adv Exp Med Biol 2010;686:349–64. 20824455 10.1007/978-90-481-9485-8_20

[2] Fryer A . Genetics of fetal anomalies. In: Kumar B , Alfirevic Z , eds. Fetal Medicine (Royal College of Obstetricians and Gynaecologists Advanced Skills). Cambridge: Cambridge University Press, 2016.

[3] Callaway JL , Shaffer LG , Chitty LS , Rosenfeld JA , Crolla JA . The clinical utility of microarray technologies applied to prenatal cytogenetic diagnosis in the presence of a normal conventional karyotype: a review of the literature. Prenat Diagn 2013;33:1119–23. 23983223 10.1002/pd.4209 PMC4285999

[4] Hillman SC , McMullan DJ , Hall G , Togneri FS , James N , Maher EJ , et al. Use of prenatal chromosomal microarray: prospective cohort study and a systematic review and meta‐analysis of the literature. Ultrasound Obstet Gynecol 2013;41:610–20. 23512800 10.1002/uog.12464

[5] Gergev G , Mate A , Zimmermann A , Rarosi R , Sztriha L . Spectrum of neurodevelopmental disabilities: a cohort study in Hungary. J Child Neurol 2015;30:344–56. 24868008 10.1177/0883073814532543

[6] Talkowski ME , Ordulu Z , Pillalamarri V , Benson CB , Blumenthal I , Connolly S , et al. Clinical diagnosis by whole‐genome sequencing of a prenatal sample. N Engl J Med 2012;367:2226–32. 23215558 10.1056/NEJMoa1208594 PMC3579222

[7] Choy KW , Wang H , Shi M , Chen J , Yang Z , Zhang R , et al. Prenatal diagnosis of fetuses with increased nuchal translucency by genome sequencing analysis. Front Genet 2019;10:761. 31475041 10.3389/fgene.2019.00761 PMC6706460

[8] Dong Z , Zhang J , Hu P , Chen H , Xu J , Tian Q , et al. Low‐pass whole‐genome sequencing in clinical cytogenetics: a validated approach [published correction appears in Genet Med. 2017 Jan; 19(1):129]. Genet Med 2016;18:940–8. 26820068 10.1038/gim.2015.199

[9] Petrikin JE , Willig LK , Smith LD , Kingsmore SF . Rapid whole genome sequencing and precision neonatology. Semin Perinatol 2015;39:623–31. 26521050 10.1053/j.semperi.2015.09.009 PMC4657860

[10] Yang Y , Muzny DM , Reid JG , Bainbridge MN , Willis A , Ward PA , et al. Clinical whole exome sequencing for the diagnosis of Mendelian disorders. N Engl J Med 2013;369:1502–11. 24088041 10.1056/NEJMoa1306555 PMC4211433

[11] Deciphering Developmental Disorders (DDD) Study. Large‐scale recovery of novel genetic causes of developmental disorders. Nature 2015;519:223–8. 25533962 10.1038/nature14135 PMC5955210

[12] Best S , Wou K , Vora N , Van der Veyver IB , Wapner R , Chitty LS . Promises, pitfalls and practicalities of prenatal whole exome sequencing. Prenat Diagn 2018;38:10–9. 28654730 10.1002/pd.5102 PMC5745303

[13] Yates CL , Monaghan KG , Copenheaver D , Retterer K , Scuffins J , Kucera CR , et al. Whole‐exome sequencing on deceased fetuses with ultrasound anomalies: expanding our knowledge of genetic disease during fetal development. Genet Med 2017;19:1171–8. 28425981 10.1038/gim.2017.31

[14] McMullan DJ , Eberhart R , Rinck G , et al. Exome sequencing of 406 parental/fetal trios with structural anomalies revealed by ultrasound in the UK PAGE study. Copenhagen: European Society of Human Genetics; 2017 (Abstract).

[15] Yadava SM , Ashkinadze E . Abstract 125: whole exome sequencing (WES) in prenatal diagnosis for carefully selected cases. Am J Obstet Gynecol 2017;216:S87–8.

[16] Shamseldin HE , Swaid A , Alkuraya FS . Lifting the lid on unborn lethal Mendelian phenotypes through exome sequencing. Genet Med 2013;15:307–9. 23037934 10.1038/gim.2012.130 PMC3908556

[17] Filges I , Nosova E , Bruder E , Tercanli S , Townsend K , Gibson WT , et al. Exome sequencing identifies mutations in KIF14 as a novel cause of an autosomal recessive lethal fetal ciliopathy phenotype. Clin Genet 2014;86:220–8. 24128419 10.1111/cge.12301

[18] Drury S , Boustred C , Tekman M , Stanescu H , Kleta R , Lench N , et al. A novel homozygous ERCC5 truncating mutation in a family with prenatal arthrogryposis – further evidence of genotype‐phenotype correlation. Am J Med Genet A 2014;164A:1777–83. 24700531 10.1002/ajmg.a.36506

[19] Wilbe M , Ekvall S , Eurenius K , Ericson K , Casar‐Borota O , Klar J , et al. MuSK: a new target for lethal fetal akinesia deformation sequence (FADS). J Med Genet 2015;52:195–202. 25612909 10.1136/jmedgenet-2014-102730

[20] Kan A , Au PK , Li M , et al. Exome sequencing on a family with 3 pregnancies affected by central nervous system malformation identified a novel stop mutation in WDr81. Prenat Diagn 2015; 35(Suppl 1:71):35.25118001

[21] Casey J , Flood K , Ennis S , Doyle E , Farrell M , Lynch SA . Intra‐familial variability associated with recessive RYR1 mutation diagnosed prenatally by exome sequencing. Prenat Diagn 2016;36:1020–6. 27616680 10.1002/pd.4925

[22] Romagnoli MPE , Palombo F , Bonara E , Seri M . RYR1 related congenital myopathy in two sib fetuses conceived through AID. Eur J Hum Genet 2016;24 (E‐Suppl I):26.

[23] Kooper AHW , Smeets D , Lugtenberg D , et al. Prenatal diagnosis of a rare, autosomal recessive disorder by combining genome wide array analysis and whole exome sequencing. Prenat Diag 2016;36(Suppl 1):76.

[24] Alamillo CL , Powis Z , Farwell K , Shahmirzadi L , Weltmer EC , Turocy J , et al. Exome sequencing positively identified relevant alterations in more than half of cases with an indication of prenatal ultrasound anomalies. Prenat Diagn 2015;35:1073–8. 26147564 10.1002/pd.4648

[25] Vora NL , Powell B , Brandt A , Strande N , Hardisty E , Gilmore K , et al. Prenatal exome sequencing in anomalous fetuses: new opportunities and challenges. Genet Med 2017;19:1207–16. 28518170 10.1038/gim.2017.33 PMC5675748

[26] Ryan E , Friedman B , Haskins A , Barbar R , Nelson Z , Al Musafri A , et al. Whole exome sequencing in 129 fetuses with abnormal ultrasound findings. Presented at ACMG Annual Clinical Genetics Meeting, Phoenix, Arizona; 2017 [http://acmg.expoplanner.com/index.cfm?do=expomap.sess&session_id=5012/]. Accessed 19 April 2020.

[27] Sa J , Melo F , Tarelho A , et al. Broad multi‐gene panel or whole exome sequencing in malformed fetuses reveals eight definitive and one likely diagnoses in fifteen studied fetuses in prenatal setting. European Society of Human Genetics; 2017 [https://2017.eshg.org/index.php/abstracts-2/online-planner-abstract-search/]. Accessed 19 April 2020.

[28] Joset P , Wisser J , Niedrist D , et al. Mendeliome and whole exome sequencing in 60 fetuses with abnormal ultrasound revealed a diagnostic yield of 30%. European Society of Human Genetics; 2017 [https://2017.eshg.org/index.php/abstracts-2/online-planner-abstract-search/]. Accessed 19 April 2020.

[29] Lord J , McMullan DJ , Eberhardt RY , Rinck G , Hamilton SJ , Quinlan‐Jones E , et al. Prenatal exome sequencing analysis in fetal structural anomalies detected by ultrasonography (PAGE): a cohort study. Lancet 2019;393:747–57. 30712880 10.1016/S0140-6736(18)31940-8 PMC6386638

[30] Petrovski S , Aggarwal V , Giordano JL , Stosic M , Wou K , Bier L , et al. Whole‐exome sequencing in the evaluation of fetal structural anomalies: a prospective cohort study. Lancet 2019;393:758–67. 30712878 10.1016/S0140-6736(18)32042-7

[31] Boyd PA , Tonks AM , Rankin J , Rounding C , Wellesley D , Draper ES . Monitoring the prenatal detection of structural fetal congenital anomalies in England and Wales: register‐based study. J Med Screen 2011;18:2–7. 21536809 10.1258/jms.2011.010139

[32] Griffiths PD , Bradburn M , Campbell MJ , Cooper CL , Graham R , Jarvis D , et al. Use of MRI in the diagnosis of fetal brain abnormalities in utero (MERIDIAN): a multicentre, prospective cohort study. Lancet 2017;389:538–46. 27988140 10.1016/S0140-6736(16)31723-8

[33] Mellis R , Eberhardt R , Lord J , Quinlan Jones E , Rinck G , McMullan D , et al. Prenatal exome sequencing for isolated increased nuchal translucency: should we be doing it? Proceedings of the ISPD, 23rd International Conference on Prenatal Diagnosis and Therapy, Singapore. Prenat Diagn 2019 (Suppl);19. 10.1002/pd.5624

[34] Normand EA , Braxton A , Nassef S , Ward PA , Vetrini F , He W , et al. Clinical exome sequencing for fetuses with ultrasound abnormalities and a suspected Mendelian disorder. Genet Med 2018;10:74. 10.1186/s13073-018-0582-x PMC6162951 30266093

[35] Chandler N , Best S , Hayward J , Faravelli F , Mansour S , Kivuva E , et al. Rapid prenatal diagnosis using targeted exome sequencing: a cohort study to assess feasibility and potential impact on prenatal counselling and pregnancy management. Genet Med 2018;20:1430–7. 29595812 10.1038/gim.2018.30

[36] Vora NL , Gilmore K , Brandt A , Gustafson C , Strande N , Ramkissoon L , et al. An approach to integrating exome sequencing for fetal structural anomalies into clinical practice. Genet Med 2020;22:954–61. 31974414 10.1038/s41436-020-0750-4 PMC7205580

[37] Cocciadiferro D , Augello B , De Nittis P , Zhang J , Mandriani B , Malerba N , et al. Dissecting KMT2D missense mutations in Kabuki syndrome patients. Hum Mol Genet 2018;27:3651–68. 30107592 10.1093/hmg/ddy241 PMC6488975

[38] Adam MP , Hudgins L , Hannibal M . Kabuki syndrome. In: Adam MP , Ardinger HH , Pagon RA , et al., GeneReviews®. Seattle: University of Washington, Seattle; 1993.

[39] Quinlan‐Jones E , Lord J , Williams D , Hamilton S , Marton T , Eberhardt RY , et al. Molecular autopsy by trio exome sequencing and full post‐mortem examination in fetuses and neonates with prenatally identified structural anomalies. Genet Med 2019;21:1065–73. 30293990 10.1038/s41436-018-0298-8 PMC6752266

[40] Wright CF , Fitzgerald TW , Jones WD , Clayton S , McRae JF , van Kogelenberg M , et al. Genetic diagnosis of developmental disorders in the DDD study: a scalable analysis of genome‐wide research data. Lancet 2015;385:1305–14. 25529582 10.1016/S0140-6736(14)61705-0 PMC4392068

[41] Zhu X , Petrovski S , Xie P , Ruzzo EK , Lu Y‐F , McSweeney KM , et al. Whole‐exome sequencing in undiagnosed genetic diseases: interpreting 119 trios. Genet Med 2015;17:774–81. 25590979 10.1038/gim.2014.191 PMC4791490

[42] ClinVar [www.ncbi.nlm.nih.gov/clinvar/]. Accessed 18 July 2020.

[43] Human gene mutation [www.hgmd.cf.ac.uk/ac/index.php]. Accessed 18 July 2020.

[44] Hillman SC , Skelton J , Quinlan‐Jones E , Wilson A , Kilby MD . “If it helps...” the use of microarray technology in prenatal testing: patient and partners reflections. Am J Med Genet A 2013;161A:1619–27. 23696517 10.1002/ajmg.a.35981

[45] Bernhardt BA , Soucier D , Hanson K , Savage MS , Jackson L , Wapner RJ . Women's experiences receiving abnormal prenatal chromosomal microarray testing results. Genet Med 2013;15:139–45. 22955112 10.1038/gim.2012.113 PMC3877835

[46] Quinlan‐Jones E , Hillman SC , Kilby MD , Greenfield SM . Parental experiences of prenatal whole exome sequencing (WES) in cases of ultrasound diagnosed fetal structural anomaly. Prenat Diagn 2017;37:1225–31. 29049852 10.1002/pd.5172

[47] Kalia SS , Adelman K , Bale SJ , Chung WK , Eng C , Evans JP , et al. Recommendations for reporting of secondary findings in clinical exome and genome sequencing, 2016 update (ACMG SF v2.0): a policy statement of the American College of Medical Genetics and Genomics [published correction appears in Genet Med. 2017 Apr; 19(4):484]. Genet Med 2017;19:249–255. 27854360 10.1038/gim.2016.190

[48] Amor DJ , Chitty LS , Van den Veyver IB . Current controversies in prenatal diagnosis 2: the 59 genes ACMG recommends reporting as secondary findings when sequencing postnatally should be reported when detected on fetal (and parental) sequencing [published online ahead of print, 2020 Feb 24]. Prenat Diagn 2020; 10.1002/pd.5670 32091628

[49] Auerbach AD , Sagi M , Adler B . Fanconi anemia: prenatal diagnosis in 30 fetuses at risk. Pediatrics 1985;76:794–800. 4058989

[50] D'Andrea A , Grompe M . The Fanconi anaemia/BRCA pathway. Nat Rev Cancer 2003;3:23–34. 12509764 10.1038/nrc970

[51] Horn R , Parker M . Opening Pandora's box?: ethical issues in prenatal whole genome and exome sequencing. Prenat Diagn 2018;38:20–5. 28695688 10.1002/pd.5114 PMC5836985

[52] Horn R , Parker M . Health professionals' and researchers' perspectives on prenatal whole genome and exome sequencing: ‘We can't shut the door now, the genie's out, we need to refine it’. PLoS One 2018;13:e0204158. 30240445 10.1371/journal.pone.0204158 PMC6150486

[53] Richardson A , Ormond KE . Ethical considerations in prenatal testing: genomic testing and medical uncertainty. Semin Fetal Neonatal Med 2018;23:1–6. 29033309 10.1016/j.siny.2017.10.001

[54] Mone F , O'Connor C , Hamilton S , et al. Evolution of a prenatal genetic clinic – 51, A 10‐year cohort study. Prenat Diagn 2020;40:618–25. 32037575 10.1002/pd.5661

[55] Costain G , Jobling R , Walker S , Reuter MS , Snell M , Bowdin S , et al. Periodic reanalysis of whole‐genome sequencing data enhances the diagnostic advantage over standard clinical genetic testing. Eur J Hum Genet 2018;26:740–4. 29453418 10.1038/s41431-018-0114-6 PMC5945683

[56] Chitty LC . Optimising exome PREnatal sequencing services (Express Study) [www.fundingawards.nihr.ac.uk/award/NIHR127829]. Accessed on 25 April 2020.

[57] Meng L , Pammi M , Saronwala A , Magoulas P , Ghazi AR , Vetrini F , et al. Use of exome sequencing for infants in intensive care units: ascertainment of severe single‐gene disorders and effect on medical management. JAMA Pediatr 2017;171:e173438. 28973083 10.1001/jamapediatrics.2017.3438 PMC6359927

[58] Illsinger S , Das AM . Impact of selected inborn errors of metabolism on prenatal and neonatal development. IUBMB Life 2010;62:403–13. 20503433 10.1002/iub.336

[59] Sofou K , Dahlin M , Hallböök T , Lindefeldt M , Viggedal G , Darin N . Ketogenic diet in pyruvate dehydrogenase complex deficiency: short‐ and long‐term outcomes. J Inherit Metab Dis 2017;40:237–45. 28101805 10.1007/s10545-016-0011-5 PMC5306430

[60] de Koning MA , Haak MC , Adama van Scheltema PN , et al. From diagnostic yield to clinical impact: a pilot study on the implementation of prenatal exome sequencing in routine care. Genet Med 2019;21:2303–10. 30918357 10.1038/s41436-019-0499-9

[61] Schofield D , Rynehart L , Shresthra R , White SM , Stark Z . Long‐term economic impacts of exome sequencing for suspected monogenic disorders: diagnosis, management, and reproductive outcomes. Genet Med 2019;21:2586–93. 31110331 10.1038/s41436-019-0534-x

[62] Kodabuckus SS , Quinlan‐Jones E , McMullan DJ , et al. Exome sequencing for prenatal detection of genetic abnormalities in fetal ultrasound anomalies: an economic evaluation. Fetal Diagn Ther 2020;47:554–64. 31962312 10.1159/000504976 PMC7446299

[63] NHS Genomics England . Fetal anomalies (Version 1.7). Relevant disorders: R21, Fetal anomalies with a likely genetic cause. Panel types: GMS Rare Disease Virtual, GMS Panel version 1.2 (signed off on 17 February 2020) [https://panelapp.genomicsengland.co.uk/panels/478/]. Accessed xx xxx xxxx.

[64] Harrison SM , Riggs ER , Maglott DR , Lee JM , Azzariti DR , Niehaus A , et al. Using ClinVar as a resource to support variant interpretation. Curr Protoc Hum Genet 2016;89:8.16.1–23. 10.1002/0471142905.hg0816s89 PMC4832236 27037489

[65] Hill M , Lewis C , Riddington M , et al. Stakeholder views and attitudes towards prenatal and postnatal transplantation of fetal mesenchymal stem cells to treat Osteogenesis Imperfecta. Eur J Hum Genet 2019;27:1244–53. 30918362 10.1038/s41431-019-0387-4 PMC6777523

[66] Gaille M , Viot G . Prenatal diagnosis as a tool and support for eugenics: myth or reality in contemporary French Society? Med Health Care Philos 2013;16:83–91. 22814726 10.1007/s11019-012-9429-1

